# The Food Supply Prior to the Implementation of the Chilean Law of Food Labeling and Advertising

**DOI:** 10.3390/nu11010052

**Published:** 2018-12-28

**Authors:** Rebecca Kanter, Marcela Reyes, Boyd Swinburn, Stefanie Vandevijvere, Camila Corvalán

**Affiliations:** 1 Department of Nutrition, Faculty of Medicine, University of Chile, Independencia 1027, Santiago 8380453, Chile; rkanter@med.uchile.cl; 2 Institute of Nutrition and Food Technology, University of Chile, Macul, Santiago 7830490, Chile; mreyes@inta.uchile.cl; 3 Department of Epidemiology and Biostatistics, School of Population Health, University of Auckland, Auckland 1142, New Zealand; boyd.swinburn@auckland.ac.nz (B.S.); s.vandevijvere@auckland.ac.nz (S.V.)

**Keywords:** law 20.606, INFORMAS, critical nutrients, food environment, Chile

## Abstract

This study aimed to evaluate the composition of the food supply ahead of the implementation of the Chilean Law of Food Labeling and Advertising (Law 20.606) in June 2016. The INFORMAS (International Network for Food and Obesity/Non-communicable Diseases (NCDs) Research, Monitoring and Action Support) framework for monitoring the composition of the food supply was used. The Law’s initial (2016) and final (2019) limits were used to evaluate if foods would receive a “High in” warning for Calories, Sodium, Sugars and/or Saturated Fats (initial/final, solids: >350/275 kcal; >800/400 mg; >22.5/10 g; >6/4 g; liquids: >100/70 kcal; >100/100 mg; >6/5 g; >3/3 g respectively). Foods were excluded if they required reconstitution, had missing information or if total labeled energy was estimated as incorrect (*n* = 942). In February 2015 and 2016, fieldworkers photographed a purposeful sample of packaged food and beverage products (*n* = 5421 and *n* = 5479) from 6 different supermarkets in Santiago, Chile. Seven percent of foods had no added critical nutrients (*n* = 720). Two-thirds of products had critical nutrients exceeding at least one initial limit indicative of a “high in” warning. Under the final phase limits, only 17% of foods would have zero warning labels. By 2019, 10 of the 17 food and beverage categories studied are predicted to have less than half of their products without a high in sodium warning label. While 8 of the 17 categories studied are predicted to have less than half their products without a high in total sugars or a high in total calories warning label, respectively; while even fewer food and beverage categories are predicted to be without a high in saturated fat warning label. Most products will have to be reformulated to avoid at least one front-of-package warning label.

## 1. Introduction

There is a global obesity pandemic and no significant decrease in obesity was observed between 1980 and 2013 in 183 countries [1]. Chile has faced a rapid nation-wide increase in overweight and obesity prevalence over this century and is one of the world’s highest consumers of sugar-sweetened beverages [2,3]. Total sales, as well as household food expenditures, of unhealthy foods has also steadily increased since 2000 [4,5]. In 2007, the Chilean government developed the Law of Food Labeling and Advertising (Law 20.606) that was passed into law in 2012 but did not officially go into effect until June 2016 [6]. The law consists of many components related to the labeling and marketing of packaged non-natural foods, applying only to products with added “critical nutrients”; specifically, sodium, sugars, and/or saturated fat. The law specifies limits for solid (per 100 g) and liquid (per 100 mL) products based on the quantities of the critical nutrients and energy. If a food or beverage product falls under the purview of the law and has added critical nutrient or energy content above the specified limits, the law mandates that the product carry a front-of-package warning label in the form of a black hexagon that states: “High in Sodium”, “High in Sugars”, “High in Saturated fats”, or “High in Calories” in accordance with the limits surpassed. As a result, a packaged food product can have up to four “High in” front-of-package (FOP) warning labels, one for each critical nutrient and calories. Furthermore, these critical nutrient limits are stipulated in the law to be implemented in a step-wise fashion with stricter limits in both 2018 (second phase) and 2019 (final phase) (Table 1).

The law includes only packaged non-natural foods with added “critical nutrients”. There are many reasons why the Chilean government decided to promote healthier food environments by regulating “unhealthy foods” that have been previously described by Corvalán and colleagues; including, but not limited to, targeting the high exposure of children (<14 years) to the marketing of foods high in critical nutrients [7]. However, since the law was approved in the Chilean senate in 2012, the practical implications of this nutritional health policy have become even more apparent. As previously mentioned, the prevalence of excess weight gain in the entire Chilean population and its continued increase among both children and adults, respectively, is alarming. Since 2005, overweight has rapidly increased to over 11% in those less than 6 y, and to a third of those less than 14 years; while one in 11 deaths among Chilean adults is attributable to overweight and obesity [8]. Between 2010 and 2016, according to the most recent Chilean National Health Survey, the prevalence of type 2 diabetes had increased in all age groups (>15 years); most notably, to 18% in those 45–64 years and 31% in those over 65 years [2]. These nutrition-related chronic health problems reflect the diet of the Chilean population (>2 years) whereby ultra-processed food products contribute at least a third of total energy intake and over half of total added sugars intake [9]. Therefore, our study aims to elucidate the importance of FOP warning labels in helping consumers to make healthier food and beverage choices. Ideally, by being able to identify the food and beverage categories that more likely to carry products with less than four FOP warning labels, if not zero.

It is uncertain how many products will be required to display “High in” FOP warning labels and therefore how the food supply will be affected by the law. Therefore, the overall goal of this study was to describe the healthiness of the food supply, specifically in terms of the different types of food and beverage products susceptible to the FOP warning labels, in anticipation of the Chilean Law of Food Labeling and Advertising. To carry out this goal, this project had two main objectives: (1) To estimate the prevalence of foods within the “High in” limits for the initial phase of the law in 2016 and final phase in 2019 and; (2) to describe the predicted proportion of each “high in” warning label by food category for the initial phase of the law in 2016 and final phase in 2019.

## 2. Materials and Methods

### 2.1. Data collection and Management

The composition and labeling of the packaged food supply were assessed using photos of packaged food and beverage products collected from supermarkets. An agreement with the Chilean National Association for Supermarkets (ASACH, for its acronym in Spanish) was signed with the Institute of Nutrition and Food Technology (INTA, for its acronym in Spanish) of the University of Chile to collect photos from one store of each of the largest supermarket chains (*n* = 6) during February 2015 and 2016, in advance of the Chilean law coming into effect in June 2016. We included the six supermarket chains that are present in all Chilean cities, and represent 100% percent of the market share of supermarkets, in our sample [10]. Each supermarket was selected for inclusion into the study based on having the greatest variety of products per chain. Trained fieldworkers took high resolution photos (approximately 25,000 photos from 17 large food categories) with digital cameras (*Canon EOS Rebel T3*) in the supermarkets. We selected the specific food and beverage categories in which to take pictures based on the likelihood that they would be most subject to the regulation. Specifically, because products in these categories contain added ingredients that increase the content of critical nutrients (e.g., unprocessed fruits or plain rice were not included). Among each large food category (e.g., beverages), all available food groups, or subcategories, such as sugar-sweetened soft drinks, were photographed. All food items representing at least 5% of the market share of a specific food group were part of that sample, indicating that our sampling approach accounted for the most relevant foods and beverages [11].

Given the concomitant global increases in both nutrition-related chronic diseases and the amount of unhealthy foods in the food environment, a global framework (INFORMAS, International Network for Food and Obesity/Non-communicable Diseases (NCDs) Research, Monitoring and Action Support) has emerged to monitor these environments [12,13]. Each product, consisting of a variable number of photos, was entered into an electronic data management platform REDCap (Research Electronic Data Capture) [14] designed exclusively for this study based on the INFORMAS food labeling and food composition protocols [13]. The label information entered from each product consisted of information related to basic characteristics about the product (year/date photos were taken, food category, food subcategory, number of photos that pertain to the product, product barcode, number of blurry photos, number of missing photos); nutrient declarations and ingredient lists on the food label. The ingredient information entered from each product consisted of all the ingredients entered as separate variables, even if a product included more than one list of ingredients (e.g., yogurt with fruit sauce). The details of the data collection are described elsewhere [15].

### 2.2. Study Sample

Following the Chilean Law of Food Labeling and Advertising, food and beverage products with no critical nutrients added were excluded from the analysis (*n* = 720) as the law does not apply to those products. Food and beverage products were further excluded from the study sample if: reconstitution was required; they were part of a “multipack”; if the photos had missing information or if total labeled energy was estimated as incorrect (*n* = 686). In relation to the latter, products were excluded if there was more than 20% difference between total energy declaration on the label and the total energy calculated from the Atwater general factor system (Carbohydrates = 4 kcal/g; Protein = 4 kcal/g; Fat: 9 kcal/g) [16]. Thus, for this study, the study sample consisted of all products collected in 2015 (*n* = 4458) and those products that were only collected in 2016, and not collected in 2015 (*n* = 2403) to create a total study sample of food and beverage products likely to fall under the purview of the law (*n* = 6861).

### 2.3. Statistical Analysis

The initial (2016) and final (2019) nutrient and energy limits for the “High in” warning labels are shown in Table 1. Please note that only foods with added critical ingredients of sugars and/or saturated fats were assessed for the “High in Calories” warning label. The ingredients list was used to determine if the product had either of these two added ingredients.

For each food and beverage product, the median nutrient composition and inter-quartile range (IQR) for the three critical nutrients (sodium, total sugars, and saturated fats) and energy were determined. The prevalence values for products within the “High in” limits for the initial phase of the law in 2016 and the final phase of the law in 2019 were calculated. For each FOP warning label, the food and beverage categories were ranked from greatest to lowest proportion estimated to carry each type of FOP warning label.

## 3. Results

### 3.1. Study Sample: Consumer Nutrition Evironment

In a large, purposeful sample of nearly 7000 unique food and beverage products from supermarket chains in Chile, over one-third consisted of processed meats (12.8%), sweets (11.9%) or sweetened bread and baked goods (11.3%) (Table 2).

Another third was evenly distributed across the categories of: beverages; desserts and ice cream; sweetened dairy and; savory dairy (Table 2).

### 3.2. Prevalence of Foods within the “High in” Limits for the Initial and Final Phases of Law 20.606

**Figure 1:**
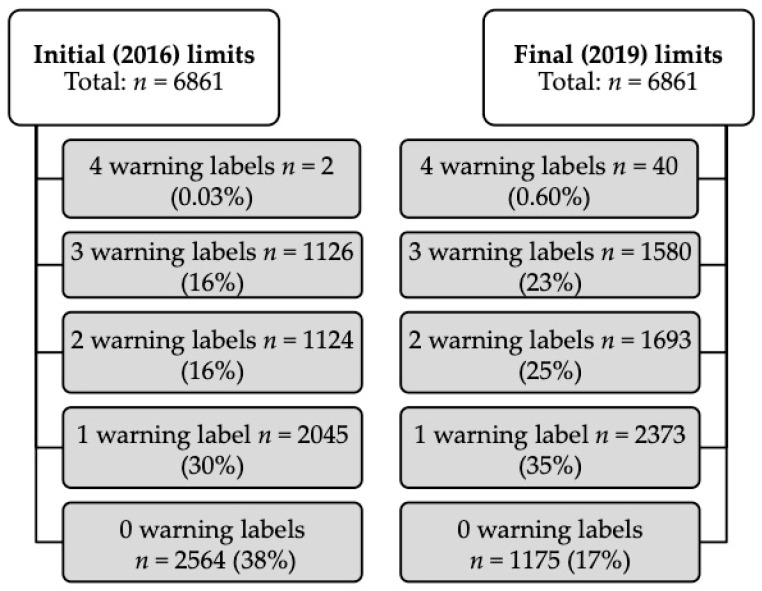
Flow chart with number of products estimated to carry between zero and all four front-of-package (FOP) warning labels considering either the initial (2016) or final (2019) limits, respectively.

The proportion of food and beverage products estimated to carry at least one FOP warning label is higher for the final phase than the initial phase because of the tighter limits (Figure 1).

Overall, the proportion of products estimated to carry zero FOP warning labels will decrease from 38% to 17% between the initial and final phases, while the proportion of products estimated to carry at least one warning label will increase from 30% to 35% (Figure 1). The 16% of products estimated to carry either two or three warning labels during the initial phase in 2016 is estimated to increase to 25% and 23%, respectively, by the final phase in 2019 (Figure 1).

### 3.3. Nutrient Composition by Food Category

The nutrient composition for each critical nutrient and energy, by food category, is summarized in Table 3. Among the 17 food categories included in this study, sodium content was highest for savory products, including: sauces and spreads; savory dairy; ready-to-eat foods and; snacks. Total sugar content was highest in sweets and; sweetened breads and baked goods. Savory dairy and sweets had the highest median levels of saturated fats. While snacks, both savory and sweet had the highest median total energy content among the 17 food categories.

### 3.4. Proportion of Each “High in” Warning Label

The proportion of each “high in” warning label by food category for the initial phase and the final phase exceeded 85% for many food and beverage categories (Table 4). Thus, 85.2% of processed meats were estimated to exceed the final limits for sodium content. Nearly all sweets (99.9%); sweetened sauces and spreads (99.6%); sweetened breads and baked goods (98.7%); processed fruits (97.3%); breakfast cereals and bars (94.6%); and desserts and ice-creams (93.4%) were estimated to exceed the final phase limits for a “High in sugars” warning label. By the final phase of the law the majority of: savory dairy (95.7%); sweetened sauces and spreads (92%); sweets (90.9%) and; sweetened breads and baked goods (86.3%) were estimated to carry a “High in saturated fats” warning label (Table 4). Under the final phase, five categories were predicted to have nearly all their products carry the FOP warning label for “High in calories”: savory snacks (100%); breakfast cereals and bars (99.6%); sweets (99%); sweetened bread and baked goods (98.1%); and sweet snacks (96.7%) (Table 4).

## 4. Discussion

Our purposeful study sample of food and beverage products in Chilean supermarkets showed that over a third was made up of processed meats, sweets and sweetened breads and baked goods. By the final phase of the law in 2019, only 17% of foods were estimated to be without any FOP “High in” warning label. Also, by 2019, most (70%) food categories were estimated to have most of their products (>80%) with at least one FOP warning label. Taken together, it is clear that there is a low prevalence of foods that contain amounts of any added critical nutrients that do not exceed the 2019 thresholds in Chile’s supermarkets. This finding that the food supply predominates in its offering of unhealthy processed food products (e.g., processed meats; sweets; and sweetened breads and baked goods) over that of “healthy” processed food products (e.g., fish and seafood products; processed fruits; processed vegetables) supports the need to identify unhealthy products through the use of FOP warning labels.

If countries are considering a similar type of governmental regulation, we recommend this type of study beforehand to identify how extensive the labeling would be. To the best of our knowledge this is the first study that has used photographic data to predict the prevalences of FOP warning labels within the consumer nutrition environment. Depending on the contextual circumstances, public policy studies may be limited by the lack of data collection prior to policy implementation. However, in the case of Chile, a window of time evolved between 2012 and 2016 in which to collect data prior to the implementation of the Chilean Law of Food Labeling and Advertising. Therefore, major strengths of this study are that it included photographic evidence of a large purposeful sample of the Chilean food supply in supermarkets and that we were able analyze food and beverage products prior to the implementation of the Chilean law. A limitation of this study is that unhealthy food categories were favored in both the sampling and analytical strategies of the food and beverage categories, which may also contribute to our findings. Ultimately, having photographic evidence of a sample of 7000 food and beverage products collected prior to the implementation of the law will be extremely useful for future studies to evaluate the impacts of the labeling on consumer purchases and reformulation.

The Chilean Ministry of Health recommends that consumers choose the food and beverage products with fewer and preferably zero warning labels [17]. Therefore, it is important to know that most of the Chilean food supply includes, and will include even greater, packaged food and beverage products with at least one FOP warning label if not more. Given these results, we now know how difficult it will be within the consumer nutrition environment for people to act according to the FOP warning label messages for two related reasons. First, the effectiveness of FOP warning labels in driving reformulation, potentially leading to fewer or zero warning labels, depends upon a consumer’s ability to use warning labels to discriminate between healthful and non-healthful products [18]; and second because health motivation has been found to be an essential factor for consumer use, and thus reaction, to warning labels [19]. It is also important to further understand what food categories appear to be most vulnerable to products always carrying at least one warning label under the final phase limits of the law; as this information will elucidate the limits of reformulation to achieve zero warning labels. We showed, for example, that under the final limits of the law 100% of savory snacks are predicted to carry the “High in calories” FOP warning label. To date, few countries have FOP warning labels mandated by a governmental regulation, but as more countries are considering similar labels the context of the food supply in these countries also remains unknown in terms of what food categories are likely to be more vulnerable to the potential FOP warnings [20].

The Chilean Law of Food Labeling and Advertising is a sound first step in trying to improve consumer education using FOP warning labels. However, major reformulation efforts will be needed to provide consumers with a wider range of healthy packaged food which carry no warning labels. In what Hawkes and colleagues refer to as a food-systems response for obesity prevention [21], reformulation, as promoted through mandatory public policies, such as Law 20.606, may be more effective than voluntary efforts [22,23]; or voluntary agreements, such as sodium reductions in the food supply in Chile [24]. How reformulation occurs in practice is often constrained by the technological challenges, and related capabilities, of industry to do so; that inherently prevent substantial reductions in nutrition content from occurring [25]. Often, food and beverage reformulation take place as a result of substituting refined or processed ingredients with other refined or processed ingredients; thereby, leading to reformulated products that are not more nutritious than their original counterpart [25,26]. At the same time, however, food and beverage product reformulation has been found to encourage healthier food choices, which may prevent mortality related to nutrition-related chronic diseases [18,27,28,29]. Without this reformulation, consumers in Chile will continue to have few healthy packaged food and beverage choices in the supermarket food supply.

## 5. Conclusions

In response to the Chilean Law of Food Labeling and Advertising, most packaged products will have to be reformulated to avoid at least one FOP warning label.

## Figures and Tables

**Table 1:** Initial and final phases front-of-package (FOP) warning label limits of law 20.606.

FOP Warning	Sodium (mg)		Sugars (g)		Saturated Fats (g)		Total Energy (g)	
	Initial	Final	Initial	Final	Initial	Final	Initial	Final
Solids,/100 g	800	400	22.5	10	6	4	350	275
Liquids,/100 mL	100	100	6	5	3	3	100	70

**Table 2:** Study sample by food category in rank order of frequency.

Food Category (*n* = 6861)	%
Processed meats (*n* = 880)	12.8
Sweets *(n* = 816)	11.9
Sweetened breads and baked goods (*n* = 775)	11.3
Beverages (*n* = 512)	7.5
Desserts and ice-creams (*n* = 502)	7.3
Sweetened dairy (*n* = 499)	7.3
Savory dairy (*n* = 486)	7.1
Savory breads and baked goods (*n* = 367)	5.4
Fish and seafood (*n* = 333)	4.9
Savory sauces and spreads (*n* = 320)	4.7
Sweetened sauces and spreads (*n* = 297)	4.3
Ready-to-eat foods (*n* = 267)	3.9
Breakfast cereals and bars (*n* = 266)	3.9
Savory snacks (*n* = 221)	3.2
Processed fruits (*n* = 206)	3.0
Processed vegetables (*n* = 84)	1.2
Sweetened snacks (*n* = 30)	0.4

**Table 3:** Nutrient composition by food category.

Food Category	Sodium (mg) Median (IQR)	Total Sugars (g) Median (IQR)	Saturated Fats (g) Median (IQR)	Energy (kcal) Median (IQR)
Beverages	11 (7.0, 18.0)	7.5 (2.7, 10.8)	0 (0.0, 0.0)	33 (14.0, 44.0)
Breads and baked goods (SV) ^1^	400 (285.2, 538.5)	3.7 (1.8, 7.2)	1.9 (0.8, 4.7)	376 (271.0, 424.0)
Breads and baked goods (SW)	197 (123.6, 289.0)	31 (25.0, 37.1)	9.6 (5.0, 13.0)	460 (400.8, 500.0)
Breakfast cereals and bars	183 (97.0, 310.0)	25 (16.6, 30.0)	2 (1.1, 4.3)	380 (368.0, 407.0)
Dairy (SV)	526.7 (333.0, 808.0)	0.8 (0.1, 3.3)	16.6 (13.5, 19.5)	324 (266.0, 357.0)
Dairy (SW)	54.7 (45.0, 65.0)	8 (6.4, 13.1)	1 (0.2, 1.4)	67 (53.0, 89.0)
Desserts and ice-creams	46 (35.0, 68.0)	17.1 (12.3, 22.0)	4.2 (2.0, 6.8)	128 (95.0, 197.0)
Fish and seafood	400.5 (306.5, 510.0)	0 (0.0, 0.2)	7.2 (3.8, 10.0)	150.5 (108.0, 196.0)
Processed fruits	8.3 (3.9, 16.0)	18 (16.0, 20.3)	14.2 (0.0, 15.0)	80 (73.0, 91.0)
Processed meats	235 (154.0, 299.0)	17.8 (8.3, 26.0)	7.2 (3.8, 10.0)	1.3 (0.3, 2.6)
Processed vegetables	290 (218.5, 433.5)	1.9 (0.2, 3.5)	5 (2.0, 6.5)	28.6 (18.0, 51.0)
Ready-to-eat foods	493 (340.0, 631.0)	1.6 (0.7, 3.1)	3.8 (1.8, 6.5)	235 (142.9, 304.0)
Sauces and spreads (SV)	571 (448.0, 800.0)	3.6 (0.4, 6.6)	11.9 (4.6, 22.8)	250 (69.0, 533.0)
Sauces and spreads (SW)	21.2 (10.0, 43.5)	51 (36.3, 57.6)	5.2 (3.2, 7.8)	245 (161.0, 309.5)
Snacks (SV)	420 (232.0, 589.0)	4.4 (1.1, 11.4)	4.2 (3.2, 6.4)	524 (488.0, 565.0)
Snacks (SW)	177 (11.9, 641.0)	5.3 (0.5, 25.0)	4.3 (2.6, 7.3)	466.5 (420.0, 496.0)
Sweets	60 (33.0, 109.5)	55 (47.6, 65.0)	16 (5.4, 20.0)	473 (384.5, 541.0)

^1^ SV denotes savory and SW denotes sweet.

**Table 4:** Proportion of foods by food group that exceed the initial and final phase limits in rank order according to the final phase limits.

Sodium (mg)	Initial	Final	Total Sugars (g)	Initial	Final	Saturated Fat (g)	Initial	Final	Total Energy (kcal)	Initial	Final
Processed meats	50.8	85.2	Sweets	98.2	99.9	Dairy (SV)	92	95.7	Snacks (SV)	97.6	100
Sauces and spreads (SV) ^1^	28.8	82	Sauces and spreads (SW)	94.8	99.6	Sauces and spreads (SW)	92	92	Breakfast cereals and bars	88.7	99.6
Dairy (SV)	27.8	67.4	Breads and baked goods (SW)	88	98.7	Sweets	89.5	90.9	Sweets	84.2	99
Ready-to-eat foods	16.2	65.1	Processed fruits	13.2	97.3	Breads and baked goods (SW)	78	86.3	Breads and baked goods (SW)	90.3	98.1
Snacks (SW)	11.8	64.7	Breakfast cereals and bars	66.1	94.6	Sauces and spreads (SV)	81.7	84.4	Snacks (SW)	93.3	96.7
Snacks (SV)	4.3	57.8	Desserts and ice-creams	72.6	93.4	Processed meats	64.4	84	Breads and baked goods (SV)	56.4	74.5
Breads and baked goods (SV)	6	50.6	Beverages	69	78.6	Processed fruits	77.8	77.8	Desserts and ice-creams	45.1	64.7
Fish and seafood	5.8	49.5	Snacks (SW)	50	78.6	Desserts and ice-creams	62.8	71.8	Dairy (SV)	16.9	53.5
Processed vegetables	1.3	29.5	Dairy (SW)	25.1	71.2	Snacks (SW)	45.8	58.3	Sauces and spreads (SV)	49.8	53.1
Breakfast cereals and bars	0.4	14.8	Snacks (SV)	15.4	44.6	Processed vegetables	43.8	56.2	Processed meats	25.2	49.6
Processed fruits	0	7.1	Dairy (SV)	10.3	27.6	Snacks (SV)	28.3	52.8	Processed vegetables	37.9	41.4
Breads and baked goods (SW)	0	5.2	Sauces and spreads (SV)	12.5	25.5	Ready-to-eat foods	34	48.3	Sauces and spreads (SW)	9.7	36
Sweets	0.4	1.9	Breads and baked goods (SV)	12.4	24.3	Breads and baked goods (SV)	25.5	39.7	Ready-to-eat foods	12.2	34.2
Desserts and ice-creams	1.3	1.3	Processed vegetables	0	10	Breakfast cereals and bars	20.1	36.2	Processed fruits	4.4	13.7
Dairy (SW)	1.1	1.1	Ready-to-eat foods	2.1	5.2	Fish and seafood	1.3	3.9	Fish and seafood	1	5.7
Beverages	0	0	Processed meats	0	0	Dairy (SW)	0	0	Dairy (SW)	0	5.7
Sauces and spreads (SW)	0	0	Fish and seafood	0	0				Beverages	0	0.3

^1^ SV denotes savory and SW denotes sweet.
